# Geostatistical analysis and mapping: social and environmental determinants of under-five child mortality, evidence from the 2014 Ghana demographic and health survey

**DOI:** 10.1186/s12889-020-09534-3

**Published:** 2020-09-18

**Authors:** Justice Moses K. Aheto, Robert Yankson, Michael Give Chipeta

**Affiliations:** 1 Department of Biostatistics, School of Public Health, College of Health Sciences, University of Ghana, P. O. Box LG13, Legon, Accra, Ghana; 2 African Institute for Mathematical Sciences, Accra-Cape Coast Road, Adisadel, Cape Coast, Ghana; 3 Malawi-Liverpool Wellcome Trust Clinical Research Programme, Blantyre, Malawi

**Keywords:** Child deaths, Under-five mortality, Geostatistical analysis, Mapping under-five mortality, Risk factors, Ghana, Sub-Saharan Africa, Developing countries

## Abstract

**Background:**

Under-five mortality (U5M) rates are among the health indicators of utmost importance globally. It is the goal 3 target 2.1 of the Sustainable Development Goals that is expected to be reduced to at least 25 per 1000 livebirths by 2030. Despite a considerable reduction in U5M observed globally, several countries especially those in sub-Saharan Africa (SSA) like Ghana are struggling to meet this target. Evidence-based targeting and utilization of the available limited public health resources are critical for effective design of intervention strategies that will enhance under-five child survival. We aimed to estimate and map U5M risk, with the ultimate goal of identifying communities at high risk where interventions and further research can be targeted.

**Methods:**

The 2014 Ghana Demographic and Health Survey data was used in this study. Geostatistical analyses were conducted on 5884 children residing in 423 geographical clusters. The outcome variable is child survival status (alive or dead). We employed a geostatistical generalised linear mixed model to investigate both measured and unmeasured child specific and spatial risk factors for child survival. We then visualise child mortality by mapping the predictive probability of survival.

**Results:**

Of the total sampled under 5 children, 289 (4.91%) experienced the outcome of interest. Children born as multiple births were at increased risk of mortality with an adjusted odds ratio (aOR) (aOR: 8.2532, 95% CI: [5.2608–12.9477]) compared to singletons. Maternal age increased risk of mortality (aOR: 1.0325, 95% CI: [1.0128–1.0527]). Child’s age (aOR: 0.2277, 95% CI: [0.1870–0.2771]) and number of children under 5 within each household (aOR: 0.3166, 95% CI: [0.2614–0.3835]) were shown to have a protective effect. Additionally, mothers with secondary education level (aOR: 0.6258, 95% CI: [0.4298–0.9114]) decreased the risk of U5M. The predicted U5M risk in 2014 was at 5.98%. Substantial residual spatial variations were observed in U5M.

**Conclusion:**

The analysis found that multiple births is highly associated with increased U5M in Ghana. The high-resolution maps show areas and communities where interventions and further research for U5M can be prioritised to have health impact.

## Background

Under-five mortality (U5M) remains a critical challenge to public health experts and the world at large because it reflects the public health and macroeconomic situations, priorities and values of every nation and the world. U5M rates are among the health indicators of utmost importance globally. It is the goal 3 target 2.1 of the Sustainable Development Goals (SDG) that is expected to be reduced to at least 25 per 1000 livebirths by 2030 [1]. Despite a considerable reduction in U5M observed globally over the past two decades, several countries especially those in sub-Saharan Africa (SSA) like Ghana are struggling to meet this target [2, 3].

The global U5M was 93 deaths per 1000 livebirths in 1990 and reduced to 39 deaths per 1000 livebirths in 2017, representing a 58% reduction though differences exist in this reduction across nations and within a given country [4]. The Global Burden of Disease (GBD) 2017 SDG Collaborators reported that several countries are on track to meet the minimum target of 25 deaths per 1000 livebirths by 2030 but noted that about 31 countries/territories need to meet yearly rates of reduction from 2015 to 2030 that are between 2 to 10 times higher than what was recorded for 1990–2015 to be able to achieve this goal [2, 3].

The rates have been persistently higher in SSA compared to other regions from 1990 to 2017, where SSA alone contributed about 50% of the global U5M in 2017, from 30% in 1990 and the rate is expected to increase to 60% by 2050. The U5M rate in SSA was seventy-nine (79) deaths per 1000 live births while that of the global rate was 41 deaths per 1000 live births in 2015 [5]. The U5M rates in SSA was 76 deaths per 1000 live births in 2017 according to the 2018 report of the United Nations Inter-agency Group for Child Mortality Estimation (UN IGME) [4, 6].

Despite the considerable reduction in U5M rates in Ghana from 127 deaths per 1000 live births in 1990 [4, 7] to 60 deaths per 1000 live births in 2014 [8], the country failed to meet the Goal 4 of the Millennium Development Goals (MDGs) targets which aimed at a two-thirds reduction in the under-five mortality rate by 2015. In addition, Ghana did not meet the under-five mortality target of 40 deaths per 1000 live births by 2015 as set in the Ghana Under-five Child Health Policy 2007–2015 [3, 8, 9].

Despite several national policies and interventions (e.g. Community-based Health Planning and Services (CHPS), Child Health Policy 2007–2015 and National Health Insurance) [3, 8, 9] rollout in Ghana to improve and promote health of children, the U5M rate remains high. In 2016, Ghana is among 8 out of 46 African countries reported to be making very little progress in reducing under-five mortality [10].

In 2017, the U5M rate was estimated at 49 deaths per 1000 live births in Ghana, with marked regional geographic inequalities [4]. Thus, the mortality rates across the country varied [3, 11], demonstrating the need for examining more localised spatial trends in U5M. Unfortunately, information on localised spatial distributions and determinants which are critical for effective design of intervention strategies that will enhance the survival of children aged below 5 years old are not readily available. We aimed to estimate and quantify under-five mortality, its localised spatial distribution, social and environmental determinants, with the ultimate goal of identifying communities at high risk where interventions and further researches can be targeted by developing risk maps of U5M. Our findings are expected to help inform health policy and intervention strategies aimed at achieving the United Nations SDG goal 3 target 2.1.

## Methods

### Study population

The 2014 Ghana Demographic and Health Survey (GDHS) dataset was used in our study [8]. The Measure DHS Program [12] provided the data which is freely available online. Data was collected on a wide range of population, health, and nutrition indicators, including geographical data. This include but not limited to data on childhood mortality, maternal and child health, use of family planning methods, household socioeconomic variables. A two-stage sample design was used to select respondents for the study. A nationally representative samples of 12,832 households from 427 clusters were selected and 11,835 eligible households were interviewed. Data were collected on 9396 women of reproductive age (15–49 years) and 4388 men aged 15–59 years. We generated data on 5884 children aged below 5 years from the interviewed women for the present study. Data on month and year of each biological child’s birth and death were extracted from complete birth histories during the survey and served as the source of identifying the number of children born in the last 5 years and child age at death. Based on data on all births to a woman within 5 years preceding the main survey, retrospective data was obtained about deceased children in the last 5 years [8].

### Outcome variable

Child survival status (dead = 1; alive = 0) was the outcome of interest in this study.

### Explanatory variables

The variables used in the analysis are as follows.

#### Child and household specific variables

Data on a child’s age – a continuous variable ranging from 0 to < 5 years preceding the survey; maternal age – a continuous variable, mother’s education – a categorical variable for highest education level attained by the child’s mother, with four categories namely no education, primary, secondary and tertiary levels, number of under 5 children in the household – a continuous non-negative variable for the number of children under the age of 5 in each household; wealth quintile – a categorical variable for the wealth index of the family, with five categories, namely poorest, poorer, middle, richer and richest; and whether a child is a twin, were obtained from DHS for all sampled children under 5 years. Detailed description of child/household level potential covariates explored in this study are presented in supplementary material Table S1 online. Wealth indices from DHS data are constructed using principal component analysis on household property ownership. Considered property include television, radio, watch, vehicles, agricultural land, type and number of livestock, bank account, materials used for house construction, access to water and sanitation facilities.

#### Community-wide and environmental variables

For each sampled cluster, we obtained data on altitude (digital elevation model - DEM) measured in meters above sea level (masl), proximity to major water bodies such as ocean, lakes and big rivers, measured in kilometres and measure of greenness (EVI), which is a proxy for rainfall and environmental suitability of disease vectors such as mosquitoes.

### Statistical analysis

a Model formulation

**Fig. 1:**
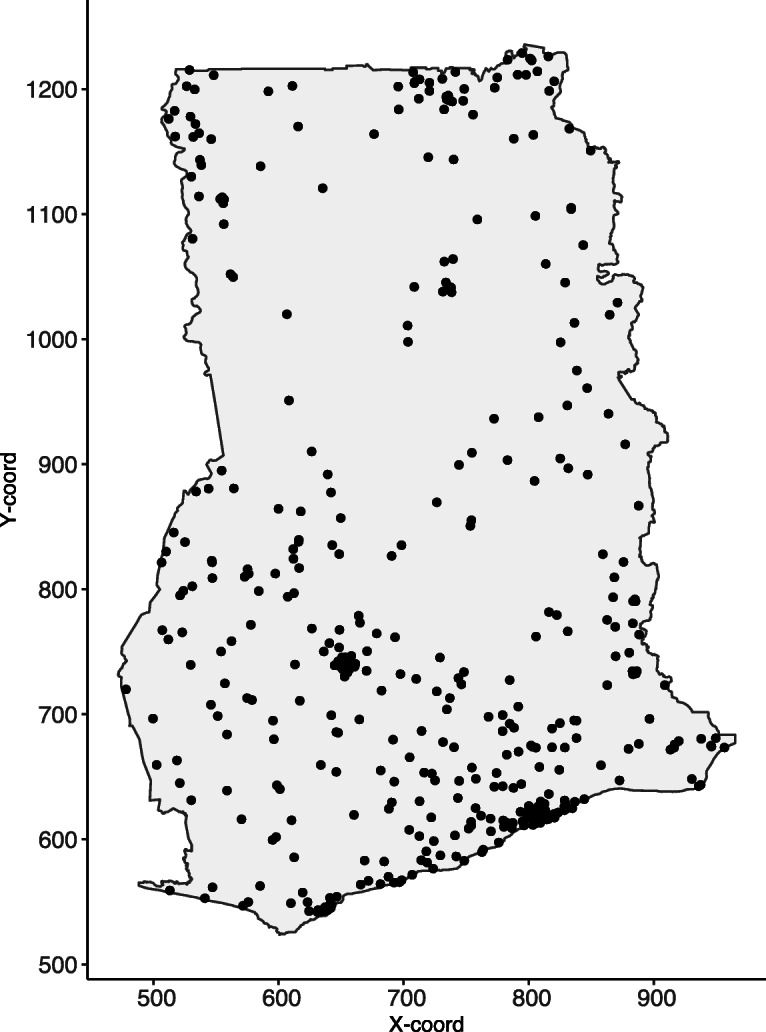
Child mortality in Ghana. The locations for clusters where DHS surveys were conducted in Ghana in 2014. Note: This map was produced by the authors using the free open software R

The data are obtained from 5884 children in each of the 423 clusters in Ghana as shown in Fig. 1. Let *i* and *j* denote the indices of the *i*^*th*^ cluster and *j*^*th*^ child within the sampled cluster. At each sampled cluster, the primary interest was survival of the *j*^*th*^ child with the outcome dead (1) or alive (0), resulting in the data-format expressed as

1 $$ Data=\left\{\left({x}_{ij},{y}_{ij},{n}_{ij}\right):{x}_{ij}\in G,j=1,\dots, {n}_i,i=1,\dots, N\right\} $$

where *x*_*ij*_ is the location of the *j*^*th*^ of *n*_*i*_ children, *n*_*ij*_ is the number of children at location *x*_*ij*_ and *y*_*ij*_ is the number of under five children that died at location *x*_*ij*_. In order to deliver valid inferences on the regression coefficients, we need to account for spatial effects. Model based geostatistics (MBG), among the many available techniques, provides a mechanism for incorporating both explained and unexplained (residual) spatial variation in the child survival outcome and allows us to predict child mortality throughout the region of interest *G*.

For the *j*^*th*^ child in *i*^*th*^ cluster, the response *Y*_*ij*_ is the binary indicator of survival. The associated covariates vector *d*_*ij*_ includes whether a child was a twin, number of other under five children within the household, mother’s education, mother’s age, child’s age, the family’s wealth index, proximity to water and a measure of wetness, namely the enhanced vegetation index. Note that the first set of three covariates are specific to each child observed and the last set of three covariates are common to all children within a given cluster. We distinguish between two sources of variation in the child survival; between-cluster variation, induced by spatially varying risk factors; and within-cluster variation induced by child specific characteristics. Each of these variations depend on both measured and unmeasured risk factors. To account for unexplained non-spatial variation, we define a generalised linear mixed model as follows. Let *S*(*x*_*i*_) denote a Gaussian process and *U*_*i*_ denote cluster specific-random effects, which are mutually independent, with mean 0 and common variance *υ*^2^. Conditionally on *S*(*x*_*i*_) and on the *U*_*i*_, the *Y*_*ij*_ are then modelled as independent Bernoulli variates with success probabilities *p*_*ij*_ given by

2 $$ \mathit{\log}\left\{\frac{p_{ij}}{\left(1-{p}_{ij}\right)}\right\}={\beta}_0+\beta \ast d{\left({x}_{ij}\right)}^T+{U}_i+S\left({x}_i\right) $$

where *d*(*x*_*ij*_) is a vector of explanatory variables associated with regression coefficients *β* for *x*_*ij*_. The spatially structured residuals *S*(*x*) are modelled as zero-mean stationary and isotropic Gaussian process with variance *σ*^2^ and correlation function

3 $$ \rho (u)= corr\left\{S(x),S\left({x}^{\prime}\right)\right\} $$

where *u* is the Euclidean distance between locations *x* and *x*^′^. We assume that *ρ*(*u*) is monotone non-increasing in distance *u*, with scale parameter *φ* that controls the rate at which the correlation approaches 0 with increasing distance *u*. Diggle (2007) outlines various parametric families for *ρ*(*u*), in the current analysis, we use the Mat $\overset{\acute{\mkern6mu}}{\mathrm{e}}$ rn class of correlation function [13], given by

4 $$ p\left(u;\varphi, \kappa \right)={\left\{{2}^{\kappa -1}\Gamma \left(\kappa \right)\right\}}^{-1}{\left(\frac{u}{\varphi}\right)}^{\kappa }{\kappa}_{\kappa}\left(\frac{u}{\varphi}\right),u>0 $$

where *φ* > 0 is the scale parameter and *κ*_*κ*_(.) is the modified Bessel function of the second order *κ* > 0. The shape parameter *κ* determines the smoothness of *S*(*x*), in the sense that *S*(*x*) is *κ* − 1 times mean-square differentiable.

b. Model validation

The model was validated by testing evidence against the residual spatial correlation in the data through the following variogram-based validation procedure (Giorgi et al., 2018). We simulate 1000 empirical variograms under the fitted model and then use these to compute 95% confidence intervals at any given spatial distance of the variogram. If the empirical variogram obtained from the data falls within the 95% tolerance bandwidth, we conclude that the adopted spatial correlation function is compatible with the data. If, instead, that falls outside the 95% tolerance bandwidth, then the data show evidence against the fitted model.

All the analyses in this study, including the maps produced were implemented in the free open software R version 3.6.1 [14].

## Results

### Sample descriptive characteristics

A total of 5884 children were sampled from 423 unique locations or clusters in the 2014 DHS survey, see Fig. 1. The average number of children per cluster varied widely, with lowest number of 1 and highest number of 59 and a median of 13 children. The locations of the sampled clusters are shown in Fig. 1. Out of the 5884 children in the dataset, 289 (4.91%) were reported dead. Of the total, 5597 (95.1%) were born singletons and 3066 (52.1%) were male children. A majority of children, 2409 (40.9%) were born to mothers with secondary education while 2042 (34.7%) belonged to mothers with no education. About 1886 (32.1%) of the children came from poorest households and 728 (12.4%) of the children came from well-endowed households. A majority of the children, 3540 (60.2%), came from rural areas. The average age of children was 1.85 years with a standard deviation 1.43; the average age for mothers was 30.6 years with a standard deviation 6.89 (Table 1).

**Table 1:** Proportions of Mortality among children under-five with respect to covariates under consideration

	Total	Alive	Dead	*P*-value
	*n* = 5884	*n* = 5595	*n* = 289	
**Child’s age** [Mean (*SD*)]	1.85 (1.43)	1.93 (1.41)	0.30 (0.78)	<.001^a^
**Mother’s age** [Mean (*SD*)]	30.60 (6.89)	30.56 (6.88)	31.36 (7.14)	0.055^a^
**U5 child in HH** [Mean (*SD*)]	1.74 (0.94)	1.76 (0.92)	1.22 (1.10)	<.001^a^
**Child’s gender**				0.231^b^
Female	2818 (100%)	2690 (95.5%)	128 (4.5%)	
Male	3066 (100%)	2905 (94.7%)	161 (5.3%)	
**Twin**				<.001^b^
Singletons	5597 (100%)	5357 (95.7%)	240 (4.3%)	
Multiple	287 (100%)	238 (82.9%)	49 (17.1%)	
**Mother’s education level**				0.109^b^
No education	2042 (100%)	1923 (94.2%)	119 (5.8%)	
Primary	1209 (100%)	1156 (95.6%)	53 (4.4%)	
Secondary	2409 (100%)	2304 (95.6%)	105 (4.4%)	
Tertiary	224 (100%)	212 (94.6%)	12 (5.4%)	
**Wealth quintile**				0.849^b^
Poorest	1886 (100%)	1789 (94.9%)	97 (5.1%)	
Poorer	1304 (100%)	1235 (94.7%)	69 (5.3%)	
Middle	1083 (100%)	1033 (95.4%)	50 (4.6%)	
Richer	883 (100%)	842 (95.4%)	41 (4.6%)	
Richest	728 (100%)	696 (95.6%)	32 (4.4%)	
**Residence**				0.938^b^
Rural	3540 (100%)	3365 (95.1%)	175 (4.9%)	
Urban	2344 (100%)	2230 (95.1%)	114 (4.9%)	
**Region**				0.002^b^
Ashanti	599 (100%)	560 (93.5%)	39 (6.5%)	
Brong Ahafo	653 (100%)	628 (96.2%)	25 (3.8%)	
Central	603 (100%)	572 (94.9%)	31 (5.1%)	
Eastern	545 (100%)	514 (94.3%)	31 (5.7%)	
Greater Accra	460 (100%)	447 (97.2%)	13 (2.8%)	
Northern	902 (100%)	842 (93.3%)	60 (6.7%)	
Upper East	551 (100%)	534 (96.9%)	17 (3.1%)	
Upper West	508 (100%)	475 (93.5%)	33 (6.5%)	
Volta	481 (100%)	459 (95.4%)	22 (4.6%)	
Western	582 (100%)	564 (96.9%)	18 (3.1%)	

^a^:*p*-value for independent *t*-test; ^b^: *p*-value for Pearson’s Chi-Square test

For children who died, majority were those born in multiples i.e. twins (17.1%) as compared to those born singletons (4.3%). Children from uneducated women experienced the highest proportion of child-mortality at 5.8%, followed by children from mothers with tertiary level education (5.4%), but it should be noted that the total sample for children born to mothers with tertiary level of education is small. Poorest and poorer households were observed to have the most deaths experienced at 5.1 and 5.3% respectively. The well-endowed households experienced the least child mortality at 4.4%. There was no difference in child mortality between rural and urban areas, with mortality at 4.9% in each of these settings. A high proportion of child deaths were observed in Northern region (6.7%), both Ashanti and Upper West at (6.5%), followed by Eastern region at (5.7%). The least proportion of child deaths were observed in Greater Accra region at 2.8%, followed by Western region at 3.1%. Infants experienced the highest mortality proportion as compared to older children; mean age for children who died was 0.3 years, standard deviation 0.78 whereas mean age for children that were still alive was 1.93 years with standard deviation 1.41 (Table 1).

### Non-spatial analysis

#### Risk factors associated with under-five mortality

For each child, the variable of interest was the binary indicator of survival (dead or alive). Selected determinants of child-mortality were estimated, and with associated 95% confidence intervals of both crude odds ratios (OR) and adjusted odds ratios (aOR). The results in Table 2 indicate that child’s age is associated with decreased odds of child mortality, with older children less likely to die, OR 0.2458 (aOR: 0.2284, 95% CI: [0.1878, 0.2778]). On the other hand, children from older mothers were more likely to die, OR 1.0167 (aOR: 1.0327, 95% CI: [1.0131, 1.0526]). Children from households that had other under five children were less likely to die, OR 0.4384 (aOR: 0.3194, 95% CI: [0.2641, 0.3864]). However, children who were born as multiple births (i.e. twins) had increased risk of child mortality, OR 4.5955 (aOR: 8.1103, 95% CI: [5.1978, 12.6549]). Mother’s education was associated with decreased odds of child mortality, children from mothers with primary level education compared to those with no education had OR 0.7409 (aOR: 0.6794, 95% CI: [0.4586, 1.0065]), albeit not significant. Similarly, children from mothers with secondary (aOR: 0.6203; 95% CI: [0.4299, 0.8948]) and tertiary (aOR: 0.5440; 95% CI: [0.2501, 1.1833]) levels of education were less likely to die with only secondary education being significantly associated with U5M. Household wealth was also shown to be associated with decreased odds of child mortality, with children from the richer households having aOR 0.6212 (95% CI:[0.3899, 0.9899]) compared to those from poorest households. All environmental covariates considered, namely digital elevation model (DEM), enhanced vegetation index (EVI) and proximity to water showed marginal associations with child mortality (Table 2).

**Table 2:** Logistic regression and 95% confidence intervals adjusted OR estimates for under-five child mortality risk factors

Variable	OR (95% CI)	aOR (95% CI)	P-wald’s Test	PLR Test
**Child’s age**	0.2458 (0.2033,0.2971)	0.2284 (0.1878,0.2778)	< 0.001	< 0.001
**Mother’s age**	1.0167 (0.9997,1.0341)	1.0327 (1.0131,1.0526)	< 0.001	< 0.001
**U5 children**	0.4384 (0.3713,0.5175)	0.3194 (0.2641,0.3864)	< 0.001	< 0.001
**Twin:** *Ref. = Singletons*				< 0.001
Multiple	4.5955 (3.2921,6.4149)	8.1103 (5.1978,12.6549)	< 0.001	
**Mother’s education:** *Ref. = No education*				0.05685
Primary	0.7409 (0.5317,1.0324)	0.6794 (0.4586,1.0065)	0.05388	
Secondary	0.7364 (0.5626,0.964)	0.6203 (0.4299,0.8948)	0.01064	
Tertiary	0.9147 (0.4968,1.684)	0.544 (0.2501,1.1833)	0.12467	
**Wealth quintile:** *Ref. = Poorest*				0.28713
Poorer	1.0304 (0.7505,1.4149)	0.8053 (0.541,1.1987)	0.28604	
Middle	0.8927 (0.6293,1.2664)	0.6923 (0.448,1.0699)	0.09779	
Richer	0.8981 (0.6178,1.3056)	0.6212 (0.3899,0.9899)	0.0452	
Richest	0.848 (0.5633,1.2764)	0.6481 (0.3712,1.1316)	0.12724	
**Elevation**	1.0001 (1,1.0002)	1.0001 (1,1.0002)	0.04274	0.057
**EVI**	1 (0.9999,1.0001)	1 (0.9999,1.0002)	0.80514	0.80509
**Water proximity**	1.0001 (1,1.0002)	1.0001 (1,1.0002)	0.15599	0.15413

#### Geostatistical analysis

**Fig. 2:**
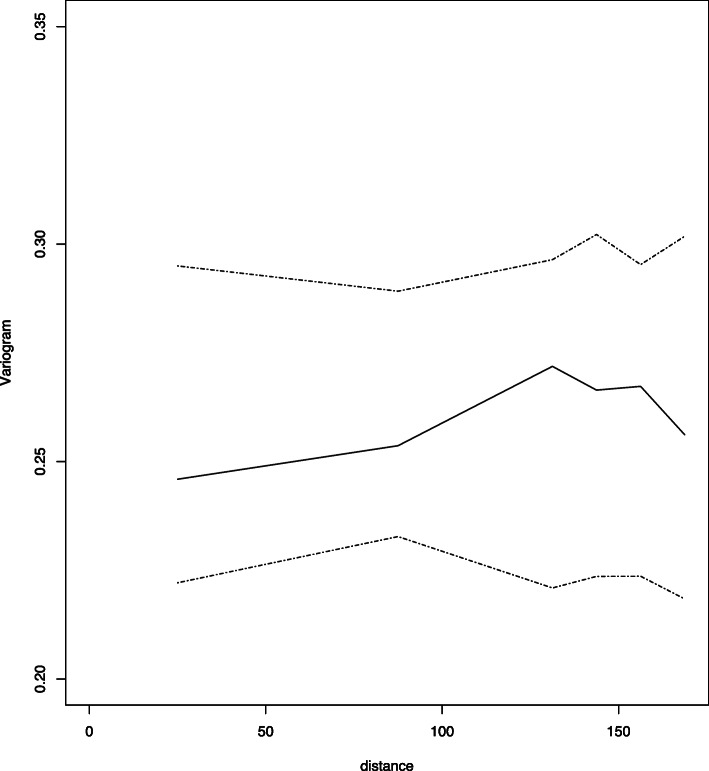
Child mortality in Ghana. Results from variogram diagnostic check for compatibility of data with the fitted geostatistical model. The solid line is the empirical variogram of the data. The dashed lines are the 95% confidence bands under the fitted spatial model. Note: This map was produced by the authors using the free open software R

In order to understand the spatial distribution of U5M and identify communities at high risk where interventions can be targeted, we implemented a Generalised Linear Geostatistical Model (GLGM) defined in eq. (2) by Monte Carlo maximum likelihood and developed a risk map of U5M. The results of testing the validity of the adopted spatial structure, showed that the empirical semi-variogram was within the 95% tolerance intervals (Fig. 2). Thus, the child-mortality data does not show evidence against the fitted geostatistical model. The results of the GLGM are presented in Table 3. The parameters *σ*^2^ and *φ* are the variance of the gaussian process *S*(*x*) and the scale of the spatial correlation *ρ*(*u*) in (kilometres), respectively. Results from GLGM revealed that maternal age (aOR: 1.0325, 95% CI: [1.0128–1.0527]), being a born as multiple births (i.e. twins) (aOR: 8.2532, 95% CI: [5.2608–12.9477]) were positively associated with U5M. Increase in child’s age (aOR: 0.2277, 95% CI: [0.1870–0.2771]), number of children aged below 5 years in households (aOR: 0.3166, 95% CI: [0.2614–0.3835]), and maternal secondary education (aOR: 0.6258, 95% CI: [0.4298–0.9114]) were shown to be associated with decreased risk of U5M.

**Table 3:** Monte Carlo maximum likelihood estimates and 95% confidence intervals for the GLGM fitted to 2014 DHS under-five mortality data

Variable	Estimate	Standard Error	95% Confidence Interval
**Intercept**	−0.577616	0.504443	(−1.566305, 0.411073)
**Child’s age**	−1.479790	0.100185	(−1.676150, − 1.283430)***
**Mother’s age**	0.032018	0.009849	(0.012715, 0.051321)***
**U5 child in HH**	−1.150036	0.097724	(−1.341570, −0.958501)***
**Twin:** *Ref. = Singletons*			
Multiple	2.110605	0.229754	(1.660295, 2.560914)***
**Mother’s education:** *Ref. = No education*			
Primary	−0.356383	0.204629	(−0.757447, 0.044682)
Secondary	−0.468651	0.191779	(−0.844530, − 0.092772)*
Tertiary	−0.595888	0.401036	(−1.381904, 0.190127)
**Wealth quintile:** *Ref. = Poorest*			
Poorer	−0.212452	0.205782	(−0.615778, 0.190874)
Middle	−0.358440	0.226691	(−0.802747, 0.085867)
Richer	−0.462223	0.248172	(−0.948631, 0.024184)
Richest	−0.409530	0.301970	(−1.001381, 0.182321)
**Elevation**	0.000089	0.000052	(−0.000014, 0.000192)
**EVI**	0.000009	0.000090	(−0.000168, 0.000185)
**Water proximity**	−0.000001	0.000001	(−0.000004, 0.000001)
*σ*^2^	0.066997	5.966316	−11.626766, 11.760761
*φ*	23.162896	0.007617	23.147966, 23.177825

***Significant at 0.1% level or less; *significant at 5% level or less;

The scale parameter *φ* has units in kilometres

**Fig. 3:**
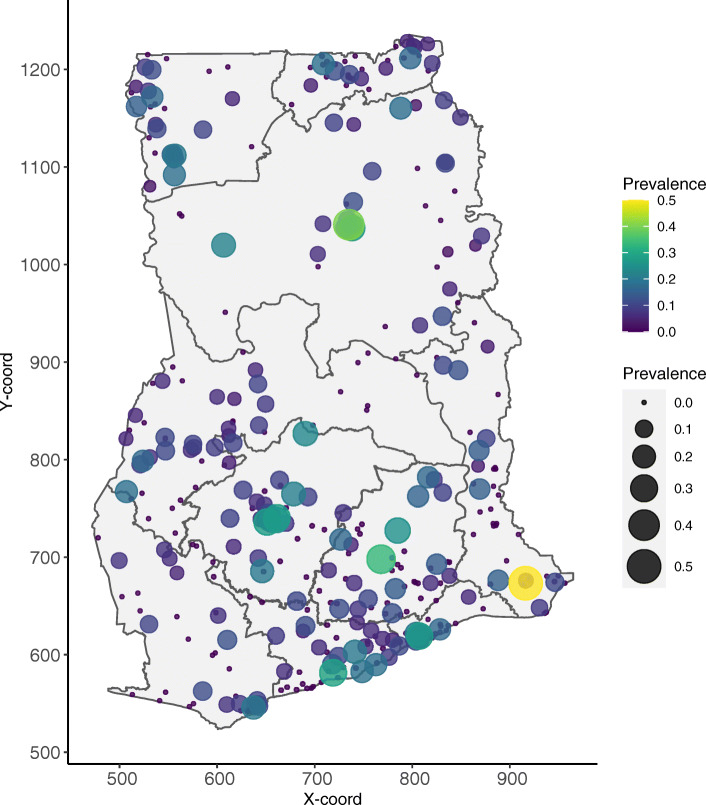
Empirical child mortality in Ghana. Child mortality prevalence from the sampled cluster locations in Ghana in 2014. The size (from small to large) and colour (from blue to yellow) of the dot represents child mortality prevalence, ranging from low to high, respectively. Note: This map was produced by the authors using the free open software R

**Fig. 4:**
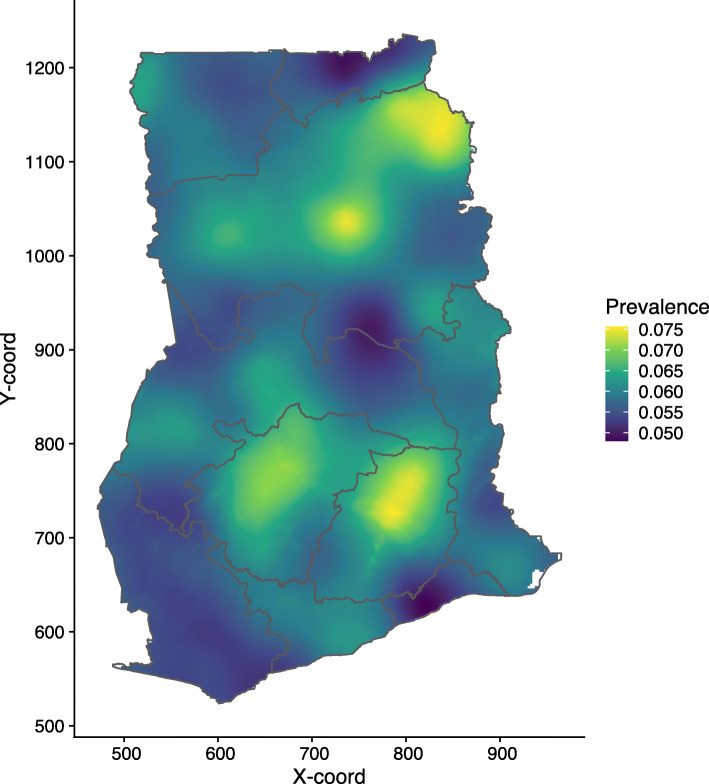
Child mortality in Ghana. Predicted child mortality risk map in Ghana showing relatively low mortality on average with pockets of high mortality in parts of Northern, Ashanti and Eastern regions of the country. Note: This map was produced by the authors using the free open software R

The assembled empirical data (Fig. 3) were used in the GLGM Eq. 2 to generate the 5 × 5 km grids of mean predictions of U5M in 2014 (Fig. 4). Point level mortality ranged from 0 to 50%, with a mean of 5%. Overall, the national predicted U5M in 2014 is low with an average of 5.98% and a median rate of 5.93%. However, this is characterised by areas with above average predicted U5M risk. These areas are mainly localised in parts of Northern, Ashanti and Eastern regions of Ghana. Parts of the Greater Accra, Western and Upper East regions are showing the lowest predicted U5M risk across the entire country (Fig. 4).

**Fig. 5:**
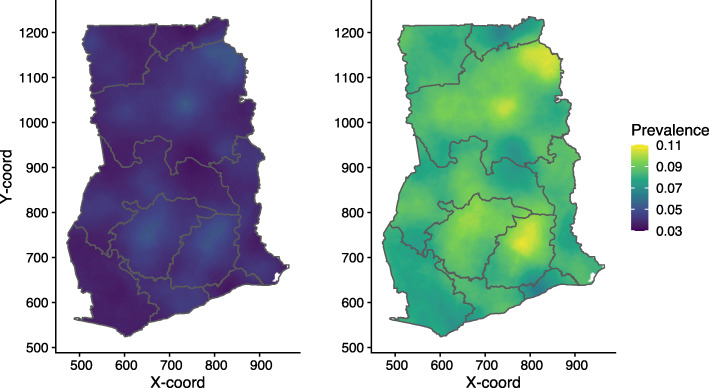
Child mortality in Ghana. Lower and upper 95% confidence maps of predicted child mortality in Ghana. Note: This map was produced by the authors using the free open software R

**Fig. 6:**
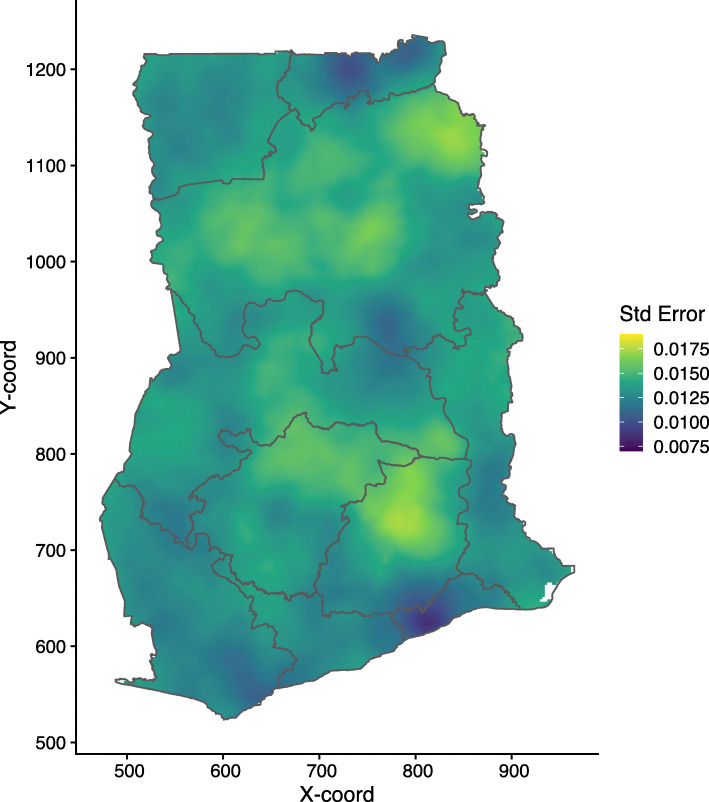
Child mortality in Ghana. Uncertainty measure for predicted child mortality. Note: This map was produced by the authors using the free open software R

In Fig. 5, we show the lower and upper quantiles maps, with a low quantile of less than 3% and a high quantile of approximately 11%. Figure 6 presents the model estimates uncertainty. The uncertainty level is relatively low, with standard errors ranging from 0.0075 to 0.0180; areas with relatively high data coverage have very low uncertainty. Relatively high uncertainty is noted mostly in Ashanti, Eastern and Northern regions of the country.

## Discussion

In our study, we set out to develop and apply Generalised Linear Geostatistical Model (GLGM) to spatially analyse under-five child mortality (U5M) in Ghana while adjusting for child, household, community and environmental factors that might be associated with U5M. Our goal was to produce spatial predictive risk maps of U5M continuously over Ghana that could help identify communities at high risk for targeted public health interventions, given the limited public health resources in the country.

Among the covariates adjusted for in the model, the study found that maternal educational level and age, number of children under five in the household, type of birth, and child’s age were associated with U5M in the spatial model (i.e. GLGM) while maternal educational level and age, number of children under five in the household, child’s age, household wealth, elevation, and type of birth were associated with U5M in the non-spatial model. However, our discussion will be based on the results from the spatial model (GLGM) because that is the focus of this study.

Broadly, our study is consistent with previous studies that examined factors associated with U5M. For example, U5M is significantly lower among children from mothers with higher levels of education [3, 15, 16]. This is in the expected direction because higher level of maternal education is likely to result in improved health seeking behaviour and utilization of health services for their offspring and themselves, and this is expected to improve the health outcomes of both the children and their mothers. It is also expected to result in optimal childcare and feeding practices with its resultant improved health outcomes for the child [3, 17–19]. Children who were born as multiple births compared to those born singleton were at increased risk of U5M. This is in line with findings from previous studies [3, 20, 21]. Monden and Smits (2017) show that mortality among under five children who were products of multiple births is 3 times higher than the mortality among singletons in sub-Saharan Africa [22]. This could partly be attributable to competition for nutrients and health complications that usually occur more among children who are products of multiple births [3, 17, 19, 23]. The unexpected finding that the number of children under-five in household is associated with lower risk of U5M warrants further investigation as reported in a previous study [3].

We used model-based geostatistics methods to map U5M risk at a fine-scale resolution of 5 × 5 km. The spatial predictive map shows that U5M risk in Ghana is at an average of 5.98% predicted U5M risk with a median of 5.92%. This is similar to the downward trend shown by both Ghana Statistical Service and The World Bank, which shows a rate of 60 and 58 deaths per 1000 live births in 2014 [7, 8], respectively. The world average at the same period shows a rate of 43.5 deaths per 1000 live births. U5M rate has continued a downward trend up to 49 deaths per 1000 live births in 2017, compared to the world rate of 39.1 per 1000 live births in the same year [7]. Thus, U5M is still a critical public health issue in Ghana.

Despite the low predicted U5M rates, there is evidence of localised high predicted U5M risk (Figs. 4–5). From Figs. 4 and 5, pockets of high U5M risk are evident especially in parts of Northern region. Furthermore, the predicted high U5M risk can also be seen in parts of Eastern and Ashanti regions.

### Strength and limitation of the study

The main strengths in this study include its representativeness and nationwide coverage allowing our findings to be generalised to the wider population of Ghanaian children under-five, and to similar populations elsewhere. Our geostatistical modelling approach permits the ability to borrow information from the sampled locations for the unsampled locations in Ghana for our predictions and mapping while simultaneously adjusting for potential confounders at the individual child, household, and community levels. In our predictions, we have also accounted for the cluster displacement which is an inherent characteristic of the Demographic and Health Survey data. The results presented here should be considered within the context of some limitations. First, we analyzed data from the 2014 DHS which is the most recent DHS cross-sectional study data in Ghana. In order to get a complete picture, a trend of U5M in Ghana should be analyzed. However, the available data cannot permit this type of analysis. This therefore requires collection and collation of more longitudinal data to allow U5M trend analysis. Secondly, secondary data from Measure DHS survey database were used and analyzed. The database had limited variables, especially on environmental and climatic factors that could have been included in the analysis to improve the understanding of U5M.

## Conclusion

The current analysis of the 2014 GDHS data set has shown that multiple births has negative association with under five children survival. The analysis has also shown that maternal education, and number of children under five are associated with reduced risk of U5M. The presented map, at 5 × 5 km resolution offers an opportunity to investigate further, especially in the highlighted regions and areas and prioritize interventions to reduce or eradicate U5M amidst available limited public health resources in developing countries like Ghana. These findings have important implications for the design of new interventions against U5M in Ghana and other similar developing countries and present new avenues for further research.

## Supplementary information

**Additional file 1: Table S1.** Definition of child variables explored

## Data Availability

Data supporting the conclusions of this article are freely available at URL: https://dhsprogram.com/data/available-datasets.cfm upon making request to the DHS Program.

## Abbreviations

DEM: Digital Elevation Model
EVI: Enhanced Vegetation Index
GDHS: Ghana Demographic and Health Survey
GLGM: Generalised Linear Geostatistical Model
MBG: Model Based Geostatistics
MASL: Meters Above Sea Level
MDGs: Millennium Development Goals
SDG: Sustainable Development Goal
U5M: Under 5 Mortality
