# Supplementary material for: Geostatistical analysis and mapping: social and environmental determinants of under-five child mortality, evidence from the 2014 Ghana demographic and health survey
Source: BMC Public Health. 2020 Sep 18;20:1428. doi: 10.1186/s12889-020-09534-3 (PMC7501707; doi:10.1186/s12889-020-09534-3)
Supplement: Supplementary file 1 — Additional file 1: Table S1. Definition of child variables explored [file 12889_2020_9534_MOESM1_ESM.docx]

| **Variable** | **Categorization** | **Description** |
| --- | --- | --- |
| Child’s age | Continuous | Child’s age in years at time of the survey |
| Sex | Categorical (female=1; male=2) | Sex of child |
| Birth order | Discrete | Birth order of the child in the family |
| Size of child at birth | Categorical (large/average=1; small=2) |  |
| Sex of HH | Categorical (female=1; male=2) | Sex of household head |
| Total child | Discrete | Total number of children in the family |
| Births 5yrs | Discrete | Number of births within the past 5 years |
| U5Child | Discrete | Number of children under the age of 5 in the family |
| HH size | Discrete | Household size |
| Mothers age | Continuous | Maternal age in years |
| HH age | Continuous | Household head age in years |
| Births in last 5 years | Discrete | Number of births in the last 5 years |
| Delivery place | Categorical (health facility=1; outside health facility=2) | Child’s place of delivery |
| Health insurance | Categorical (Yes=1; No=2) | Health insurance status of the mother |
| Drinking water | Categorical (piped=1; Non-piped=2) | Household source of drinking water |

Table S1 Definition of child variables explored
